# Syphilis Communicated by Vaccination

**Published:** 1863-09

**Authors:** 


### SYPHILIS COMMUNICATED BY VACCINATION.

M. Devergie read before the Academy of Medicine, May 19th, a paper entitled "Tuberculous syphilide generalized in a boy fifteen years of age, with the presumption of infection by vaccination inoculated from arm to arm, at the Hospital Saint Eugenie."

Alfred Desire S., aged fifteen years, a cabinet maker, March 11, 1863, entered St. Louis Hospital. His father is dead from wounds; his mother enjoyed good health. Seven months previously Alfred Desire was under the care of M. Barthez, at Saint Eugenie, for a pleurisy. He left the Hospital, cured, at the end of twenty-three days. Eight or ten days after his entrance into Saint Eugenie, he was vaccinated on the right arm with some matter taken from the arm of a nursing child. A number of children were vaccinated the same day with the same vaccine matter. The lancet which was used had not been used before, according to the declaration of M. Fritz, the *interne* of the ward. Three days after the vaccination a small brown crust appeared on the point of insertion of the matter. The crust enlarged and the skin became red; the boy did not say anything about it, and did not have his arm examined before leaving the hospital, nor during his stay in the convalescent house. However, during this time not only the primitive redness persisted, but it extended, without any inconvenience being felt by the boy. Five or six weeks afterwards, an eruption of *boutons* on the arms and thighs; thickening of the skin around the colored point of the arm; two new eruptions of *boutons* more extended; hoarseness towards the third month; osteoscopic and rheumatic pains. On his admission into St. Louis Hospital, (March 11,) papular eruption over the whole surface; impetigo of an elliptical form on the upper lip; three tubercles of recent origin and indurated, on the prepuce; some enlargement of ganglia in left groin; in the neighborhood of the vaccination, on the right arm, a round surface where the skin is thick, hard, unequal, and of a sombre red; ganglia of the armpit voluminous and indurated on the right side only. The anus is healthy, and presents none of the characteristic signs of syphilis. Anti-syphilitic treatment (iodide potas. pills of Dupuytren) was followed by improvement at the end of six weeks; impetigo of the lips cured; all the tubercles have become more pale; the skin around the vaccination has become supple, discolored at its circumference; hoarseness has disappeared; health excellent.

The diagnosis of the disease, says M. Devergie, has not been doubtful a single moment. At present, as the symptoms are notably diminished, they have not, however, yet raised the least uncertainty on the part of the members of the Academy to whom the boy has been shown.

What is the point of departure of these accidents? There is the whole difficulty. We have not the certificate of its origin; perhaps we may be able to obtain it in consequence of the efforts on the part of the administration of the hospitals. We have not found on the penis of the boy any trace of chancre. The facts which he has given accord with that which science has taught relative to the evolution of syphilitic accidents. All these reasons establish strong presumptions in this case on the inoculation of syphilis by means of vaccination.

*M. Ricord*—Admitted that he had denied the transmissibility of syphilis by vaccination. For a long time demonstrative facts have failed, and everything justified us in denying it. But observations have multiplied, the proofs have accumulated, and the demonstration is at present so convincing that it is not permitted to any one to hesitate to accept as certain this mode of transmission. Recently still we observed in the service of M. Trousseau an analogous case to that of M. Devergie. But, in spite of all, that which is certain is, that in one way or another the syphilitic accidents were not the result and as the explosion of an anterior and latent constitutional syphilis. In two patients the infection was recent, and the vaccinal puncture had evidently served as the point of entrance of the syphilis. Nevertheless, in spite of the facts collected up to the present, a question remains still to be solved: it is that relative to the conditions in which the vaccinated persons must be found in order to transmit syphilis. Here is all the obscurity—all the confusion.

At best, what presumption can be drawn from the examinations of the parents or the child? A child born syphilitic may present at birth, and for a long time afterwards, the most perfect appearance of health. It could then at the time when it is vaccinated present no manifestation of constitutional syphilis. The father and mother might equally, however syphilitic they may be, preserve no trace of the primitive accident, and present actually no secondary sign of the disease. Then there is another difficulty—another mystery: the father according to the law, *pater quem nuptiæ demonstrant*,—is he always the father according to nature? Alas, no! It is this fact which may cause a child to be syphilitic, although its legal father may not be so; and consequently it is barely possible, in some circumstances, to assure the health of the child from that of the father.

Can we learn anything from the age of the child? No. The epoch when the constitutional symptoms of syphilis break out with children is very variable. These symptoms show themselves rarely before birth; we do not see them appear, as a general rule, before the end of six weeks or two months, or even in the fifth or sixth month. This uncertainty in the manifestations of hereditary syphilis leads us to the fact that age does not authorize us to prejudge anything.

Is it possible for us to be instructed better by the puncture and the vaccinal eruption? If in some exceptional circumstances the vaccinal *bouton* has appeared suspicious or gives place to doubt, in the immense majority of cases, as in that of M. Devergie and M. Trousseau, the vaccinal eruption offers no specific character, and has all the appearance of the best vaccination.

M. Ricord concluded from all these considerations, that very often, the oftenest even, no index could enlighten the physician on the state of the health of the child vaccinated, nor of the quality of the vaccine matter which is used; that we could not consequently place on the operator the responsibility of a transmuted syphilis by vaccination.

*M. Gosselin*—Is very ready to believe that syphilis may be transmuted by vaccination; but it is a fact so grave that he would not wish any one to pronounce it until he had proved it by all means. It would be necessary, for example, to know and examine the parents of the vaccinated child, to follow the the child, and to know what happens to all those who are inoculated with its vaccine matter.

*M. Devergie*—Said that he had commenced an inquiry of this kind, and that he relied on the well known zeal and devotion of M. Husson, director-general of *assistance publique*, to carry it out.

*M. Depaul*—Has been for a long time convinced of the contagion of secondary accidents, and especially of the transmissibility of syphilis by vaccination. Science possesses at present on this subject facts very authentic and more complete than the one reported by M. Devergie, which assuredly is not sufficient by itself to be convincing. He desired to place himself in opposition to the opinions emitted by M. Ricord. He believed that we can very essily recognize in general, and by certain signs, a child attacked by hereditary syphilis. For it is an error to pretend that the most part of syphilitic infants present at their birth, and for a long time after, the best appearance of health, and that the accidents do not break out until the age of two, three and six months. Such an opinion can only rest on an incomplete or superficial study of the question. The truth is, that constitutional syphilis manifests itself ordinarily in infants from the birth, or from the first weeks, either in the form of pemphigus neonatorum, or by other symptoms less evident, perhaps, but not less characteristic for a severe observer, for a careful *clinician*. M. Depaul did not think that he had ever been deceived, either in his hospital practice or that of the city, or in the vaccine service in which he had served with M. Bosquet. He had examined with the greatest care all the children who were brought to him by the mothers, and he declared that after this double examination he was so sure of the vaccine matter which he employs that he would not hesitate to vaccinate himself with it.

*M. Ricord*—Congratulated M. Depaul on the kind of immunity which he enjoyed from the academic vaccine matter; but in his opinion that simply proves that the transmission of syphilis by vaccination is very rare and very difficult. For what serious guaranty can be offered for all those children which are brought from all quarters to the Academy to be vaccinated? Do we know from whence they come, and who are their parents? We must, then, attribute not only to the skill of M. Depaul, but perhaps also a little to chance, the happy fact stated by the honorable vaccinator. M. Ricord has not certainly seen as many new-born children as M. Depaul; but he believes he has seen as many syphilitic ones as that gentleman. He says with great certainty that he has rarely seen constitutional syphilis manifest itself at birth, while he has often seen it, and the most frequently, not show itself until two or three months after.—*Edit. Trans. Lancet and Observer.*

---

☞ Nobody will deny, that cures may be effected in quite different ways, and that the apparent contradictions in treatment may dissolve into unity, by the various operations of the organism. Organic nature is not confined within such narrow limits as are our systems; if it were so, one after another would not have had its ascendency, and been applied with success.—*Hufeland.*

